# An unusual presentation of a depressed clavicle fracture in a child leading to venous congestion

**DOI:** 10.1308/rcsann.2025.0024

**Published:** 2025-06-17

**Authors:** AC Pillai, JT Shajan, A Varma, AA Ishaq, MZ Farook

**Affiliations:** ^1^ Dr Gray’s Hospital, NHS Grampian, UK; ^2^ University of Birmingham Medical School, UK

**Keywords:** Case report, Cervical rib, Clavicle, Fracture, Paediatric

## Abstract

Clavicle fractures are common in children, comprising up to 15% of fractures presenting to the emergency department. Typically, mid-shaft clavicle fracture in the paediatric population is managed conservatively, with surgery recommended in a select few. A 12-year-old boy presented with a depressed mid-shaft clavicle fracture with an ipsilateral cervical rib exhibiting features of venous congestion. This case was managed conservatively using a sling and the patient was admitted for overnight observation with early mobilisation encouraged. His recovery was complicated by a re-fracture 4 weeks later, which was managed conservatively with a good clinical outcome. This is the first report of such a fracture associated with an ipsilateral cervical rib. This case highlights that such injuries with vascular concerns could be managed conservatively with good outcomes.

## Background

The clavicle is one of the most common long bone fractures to occur in children, comprising up to 15% of fractures presenting to the emergency department in this population.^1^ Typically, mid-shaft clavicle fractures in the paediatric population are managed conservatively, with surgery required if it is an open fracture, if there is neurovascular compromise, a threat to skin or evidence of non-union. We present a very unusual case of a depressed mid-shaft clavicule fracture with an associated ipsilateral cervical rib exhibiting features of mild venous congestion in a 12-year-old boy.

A cervical rib is a congenital abnormality leading to an accessory rib usually developing from the seventh vertebra.^2^ This has an incidence of 0.05% to 3% and is usually asymptomatic in the vast majority of patients.^3^ In some rare occasions, it can cause thoracic outlet syndrome leading to compression of the subclavian artery, vein and/or the brachial plexus, potentially leading to permanent neurovascular sequelae.^4^ As far as we know, this is the first report of such a fracture associated with an ipsilateral cervical rib causing venous congestion.

## Case history

A 12-year-old right-hand dominant, healthy boy presented to the emergency department of our district general hospital following a fall from standing height while play-fighting with his friend. His friend grabbed his right arm and pulled him to the ground and then landed on him.

**Figure 1:**
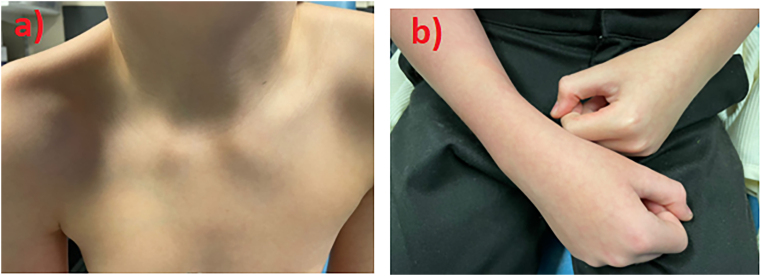
Clinical images of (a) right shoulder and (b) right forearm upon presentation in the emergency department

On assessment, his observations were largely unremarkable with a Paediatric Early Warning Score of 0. The patient had an obvious depression noted over the right clavicle region with associated generalised tenderness (Figure 1a). He had reduced range of movement of the right shoulder in all directions, likely secondary to his sustained clavicle injury. He had normal neck range of motion without tenderness to palpation of the cervical spine. His right hand appeared congested with more blanching than his left arm, with a capillary refill time (CRT) of <3s (Figure 1b), and palpable brachial and radial pulse. His right upper extremity was neurologically intact throughout the peripheral branches of the brachial plexus. His left arm was warm and well perfused with a CRT of 2s and a palpable brachial and radial pulse.

**Figure 2:**
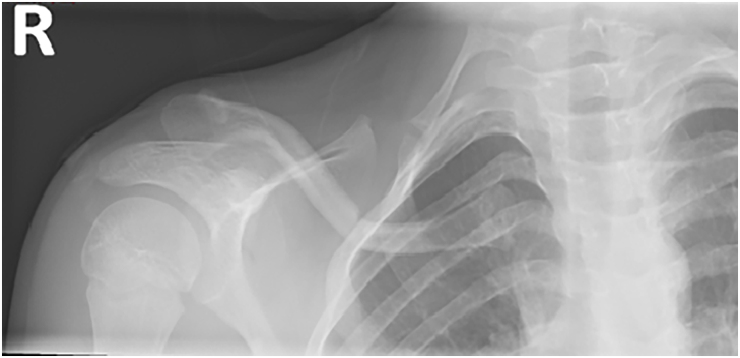
Right clavicule antero-posterior x-ray taken on initial admission to the emergency department

**Figure 3:**
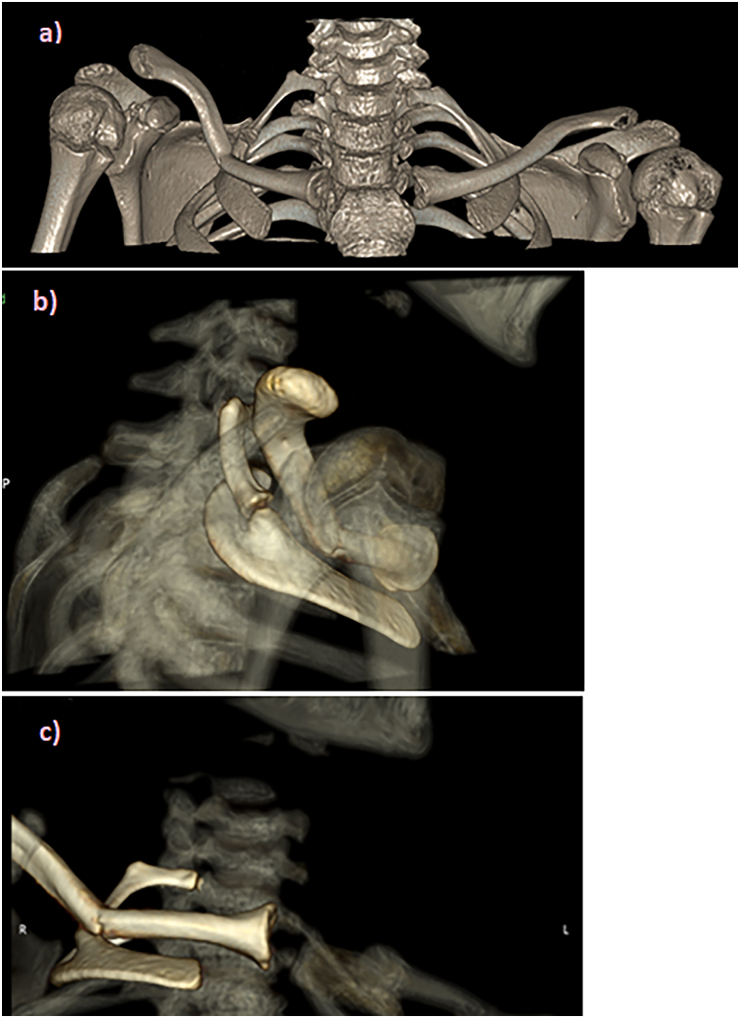
Three-dimensional computed tomography reconstruction of (a) both clavicles, (b) postero-lateral view the right clavicle and (c) anterior view of the right clavicle.

**Figure 4:**
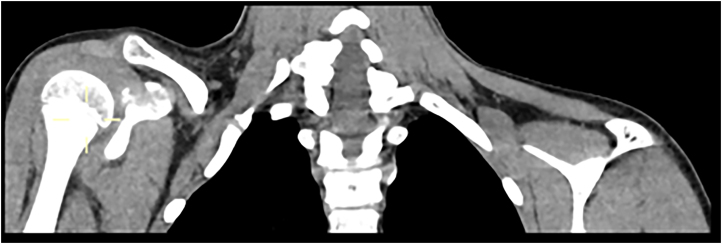
Coronal computed tomography view of the clavicle to show the extent of compression on surrounding structures

The patient underwent plain radiograph of the clavicle (Figure 2), which revealed a depressed right mid-shaft clavicle fracture and also an ipsilateral cervical rib with pseudoarthrosis. The patient was placed in a broad arm sling for comfort and given analgesia. A computer tomography scan with contrast was undertaken with three-dimensional reconstruction of his clavicle and adjacent joint; the main concern being of subclavian venous congestion (Figure 3). It was reported as a greenstick fracture of the middle third of the right clavicle with superior tilting of lateral fragment forming an inferior angle resulting in narrowing of the costoclavicular distance. The sagittal reconstructions showed that the subclavian artery, and to a lesser extent the subclavian vein, were compressed by the clavicle (Figure 4). Incidentally, as seen on the x-rays, there was a right bony cervical rib pseudo-articulating with the first rib, which was uninjured.

**Figure 5:**
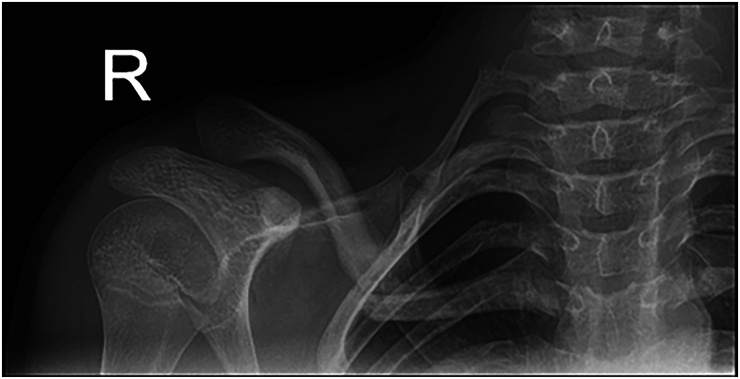
X-ray following re-injury showing a complete fracture of the right clavicle

The patient was referred to the local tertiary centre who advised overnight admission for observation of neurovascular status. The venous congestion resolved the next morning and the patient was discharged. He was advised to be in a sling immobilised for 2 weeks followed by gentle movements. At the first week follow-up, the right and left arm appeared symmetrical and a CRT of 2s was noted bilaterally. Everything was uneventful until 1 month from injury, when he again presented to our emergency unit with a further fracture of the right clavicle following another fall. The x-ray showed a healing clavicle fracture with callus formation; however, with worsening of alignment since the previous examination thus indicating a re-fracture through the same site (Figure 5). On examination there was no further worsening of symptoms and an absence of neurovascular compromise. He was advised to continue with a broad arm sling with follow-up in 1 month after the second injury. He recovered full range of movement in his shoulder and was subsequently discharged from clinic 1 month after the second injury.

The patient was happy with the treatment received and attempted to adhere to instructions given as best as he could. His family member was a healthcare worker and therefore well versed in the progress and prognosis of the condition. If anything, he did admit to resuming playground activities at the 3-week mark which made him more prone to the re-fracture.

## Discussion

Clavicle fractures constitute between 2.6% and 15% of all paediatric fractures.^1,5,6^ The clinical anatomy of the clavicle and sternocleidomastoid muscle attachment almost always displaces the medial clavicle superiorly following a fracture. Hence, neither the Neer classification nor the Rockwood classification of clavicular fracture considers inferior displacement of the clavicle.^7^ The cervical rib might play a part in preventing superior migration of the proximal part of the clavicle during mid-shaft fracture. However, in this particular case, a combination of the increased ductility of the paediatric bone, a thicker periosteum and the cervical rib might have led to inferior displacement of the clavicle.

In a review of literature, we found no other published evidence that informed us of how to manage this particular pattern with venous congestion. The closest was a case report by Fang and colleagues, who discussed a distal third clavicle fracture with an inferiorly displaced medial fragment (type 2b fracture) following a motorcycle crash accident in a 20-year-old woman.^4^ This was managed operatively because of its instability and the association with high rates of non-union, mal-union and undesirable symptoms of this fracture type.^1^ But in our report, we describe a paediatric mid-shaft, inferiorly displaced greenstick clavicle fracture with an associated ipsilateral cervical rib. Although our initial assessment showed mild vascular compromise on the child’s affected side, triggering referral to the local tertiary centre, no worsening of circulation was observed. The case was therefore managed conservatively by the paediatric orthopaedic team.

Our rationale regarding conservative management was further supported by the fact that paediatric clavicle fractures are typically treated nonoperatively because of their low rates of non-union or delayed union.^1^ Moreover, Strauss and colleagues reported that only 0.9% of clavicle fractures showed disturbed bone healing in the paediatric population taking into consideration all the locations of clavicle fractures in this population.^6^ Furthermore, surgical management had no benefit on patient-reported quality of life, satisfaction, shoulder-specific function or the prevention of complications after completely displaced clavicle shaft fractures in adolescents at up to 2 years after injury.^8^

## Conclusion

Our case is unique considering the fracture pattern, inferior displacement, associated cervical rib, transient venous congestion, clinical management and re-injury. As far as we know, no similar case has been reported in the literature that considers a clavicle fracture in the presence of an ipsilateral cervical rib and venous congestion. This highlights that such injuries can be managed conservatively with close observation in the outpatient clinic and we hope this article will inform clinicians on how to manage such injuries.
